# Cost-effectiveness and budget impact analyses of a colorectal cancer screening programme in a high adenoma prevalence scenario using MISCAN-Colon microsimulation model

**DOI:** 10.1186/s12885-018-4362-1

**Published:** 2018-04-25

**Authors:** Arantzazu Arrospide, Isabel Idigoras, Javier Mar, Harry de Koning, Miriam van der Meulen, Myriam Soto-Gordoa, Jose Miguel Martinez-Llorente, Isabel Portillo, Eunate Arana-Arri, Oliver Ibarrondo, Iris Lansdorp-Vogelaar

**Affiliations:** 1 Gipuzkoa Primary Care – Integrated Health Care Organizations Research Unit, Alto Deba Integrated Health Care Organisation, Avda Navarra 16, 20500 Arrasate-Mondragón, Gipuzkoa Spain; 2 Health Services Research on Chronic Patients Network (REDISSEC), Arrasate - Mondragón, Gipuzkoa Spain; 3 Biodonostia Health Research Institute, Donostia - San Sebastian, Gipuzkoa Spain; 4 Basque Country Colorectal Cancer Screening Programme, Basque Health Service, Bilbao, Bizkaia Spain; 5 Clinical Management Unit, Alto Deba Integrated Health Care Organisation, Arrasate - Mondragón, Gipuzkoa Spain; 6 Department of Public Health, Erasmus MC, University Medical Center, Rotterdam, The Netherlands; 7 Accounting Department, Alto Deba Integrated Health Care Organisation, Arrasate - Mondragón, Gipuzkoa Spain; 8 BioCruces Health Research Institute, Barakaldo, Bizkaia Spain

**Keywords:** Colorectal Cancer, Mass screening, Cost-effectiveness analysis, Budget impact analysis

## Abstract

**Background:**

The Basque Colorectal Cancer Screening Programme began in 2009 and the implementation has been complete since 2013. Faecal immunological testing was used for screening in individuals between 50 and 69 years old. Colorectal Cancer in Basque country is characterized by unusual epidemiological features given that Colorectal Cancer incidence is similar to other European countries while adenoma prevalence is higher. The object of our study was to economically evaluate the programme via cost-effectiveness and budget impact analyses with microsimulation models.

**Methods:**

We applied the Microsimulation Screening Analysis (MISCAN)-Colon model to predict trends in Colorectal Cancer incidence and mortality and to quantify the short- and long-term effects and costs of the Basque Colorectal Cancer Screening Programme. The model was calibrated to the Basque demographics in 2008 and age-specific Colorectal Cancer incidence data in the Basque Cancer Registry from 2005 to 2008 before the screening begun. The model was also calibrated to the high adenoma prevalence observed for the Basque population in a previously published study. The multi-cohort approach used in the model included all the cohorts in the programme during 30 years of implementation, with lifetime follow-up. Unit costs were obtained from the Basque Health Service and both cost-effectiveness analysis and budget impact analysis were carried out.

**Results:**

The goodness-of-fit of the model adaptation to observed programme data was evidence of validation. In the cost-effectiveness analysis, the savings from treatment were larger than the added costs due to screening. Thus, the Basque programme was dominant compared to no screening, as life expectancy increased by 29.3 days per person. The savings in the budget analysis appeared 10 years after the complete implementation of the programme. The average annual budget was €73.4 million from year 2023 onwards.

**Conclusions:**

This economic evaluation showed a screening intervention with a major health gain that also produced net savings when a long follow-up was used to capture the late economic benefit. The number of colonoscopies required was high but remain within the capacity of the Basque Health Service. So far in Europe, no other population Colorectal Cancer screening programme has been evaluated by budget impact analysis.

**Electronic supplementary material:**

The online version of this article (10.1186/s12885-018-4362-1) contains supplementary material, which is available to authorized users.

## Background

The cost effectiveness of colorectal cancer (CRC) screening has been widely documented [1–4]. However, specific evaluations for different programmes are necessary because local CRC epidemiology (cancer incidence and adenoma prevalence) and the actual implementation of screening (type of test, attendance rate, and surveillance schedule) can cause variations in efficiency [4–6]. CRC in Basque country is characterized by unusual epidemiological features given that CRC incidence is similar to other European countries while adenoma prevalence is higher [7]. Therefore the need for independent evaluation is especially noteworthy. As with all public health interventions, cancer screening programmes must measure their value, i.e., the health outcomes achieved per euro spent for the whole population [8, 9].

The Basque CRC Screening Programme began in 2009 at the suggestion of the Cancer Advisory Council, which took into account the significant increase of both CRC incidence and mortality in the Basque Country from 1986 through 2008 [10–12]. Like most European programmes, it uses faecal immunochemical testing (FIT) to detect microscopic bleeding from adenomas or preclinical CRC [10, 13]. Individuals with positive test results are referred for a diagnostic colonoscopy and can then be assigned to a surveillance schedule [13]. As more cohorts are included in the programme, the number of colonoscopies grows, and programme feasibility relies on its capacity to respond to that demand [3, 5, 14]. The Basque CRC epidemiology of high adenomas prevalence may constitute a challenge because it may mean that the number of colonoscopies required in the CRC screening programme will be higher. Experts have underscored the need to tailor the implementation of CRC screening in populations within the context of organized programmes [6, 15].

During the first four years of implementation, the Basque programme performed 295,934 screening tests, and 17,146 diagnostic colonoscopies were carried out to confirm positive FIT results. Such marked resource consumption alone justifies conducting an economic assessment that takes into account the results in the middle and long term [5, 9]. Besides the cost effectiveness, for a comprehensive economic evaluation, it is essential analysing the programme’s affordability regarding the global budget impact [16]. This analysis addresses the expected changes in the expenditure of a healthcare system after the adoption of a new intervention [16]. As with other CRC screening programmes, sustainability analysis must also tackle the demand for colonoscopies to avoid future inability to meet the need [2–6, 15]. To carry out the economic evaluation of cancer screening programmes, widespread use of decision models has been the rule generally, [2–6] as well as in the Basque Country [17, 18].

Because the implementation of the Basque CRC programme has been complete since 2013, decision-makers should now be informed as to whether the resources are appropriately allocated and the programme is sustainable given the high prevalence of adenomas. The object of our study was to economically evaluate the programme compared to no screening via cost-effectiveness and budget impact analyses with microsimulation models calibrated to the Basque epidemiological features of CRC incidence and adenoma prevalence [11, 19, 20].

## Methods

As the programme targeted the population between 50 and 69 years of age and used biennial FIT screening and complete colonoscopy to confirm positive results, its evaluation required follow-up of the target population at middle and long term. To estimate the economic results of the screening strategy implemented in the Basque Country, the Microsimulation Screening Analysis model for colorectal cancer (MISCAN-Colon) was applied. MISCAN-Colon is a stochastic microsimulation model for colorectal cancer (CRC) developed using Delphi programming language (Borland Software Corporation, Scotts Valley, California, United States). The aim of the model was to explain and predict CRC incidence and mortality trends, as well as, assessing the effect of primary prevention of CRC, screening for CRC, and surveillance after polypectomy in terms of both health and costs. The structure of the MISCAN-Colon model and the sources that inform the parameters of the model have been fully described in previous publications, [21–25] as well as in a standardised model profile of the Cancer Intervention and Screening Network (CISNET) [26]. A full description of the model was also included in the Model Appendix section of the Additional file 1.

Individuals of a large population were simulated using MISCAN-Colon. They were created at birth and lifelong follow-up was applied. Natural history of CRC was included according to the adenoma–carcinoma sequence [27, 28]. The model assumes the possibility for more than one adenoma at the same time in each individual. Each adenoma can independently progress in size (≤5 mm, 6–9 mm, ≥10 mm) and develop into CRC. In addition, some will become malignant, transforming to stage I CRC; some cases of CRC may even progress to more advanced stages. At any stage, CRC may be diagnosed due to the development of symptoms. When CRC finally develops, the survival rate after diagnosis depends on the stage at which the cancer was detected. In addition, at any time during the individual’s life, the process may be interrupted by his/her death from other causes. With this model, the entire target population can be followed from birth to death to measure both the long-term costs and health outcomes related to the programme.

### Simulated population

We reproduced the entire Basque population in 2008: 2,230,000 people, 51% women. The screening programme begun in 2009 was fully implemented by 2013. In order to reproduce the implementation strategy, the population was divided into different strata based on its age structure and the calendar year at which individuals were invited into the programme for the first time (or never invited in the event that they were 70 or older in 2009). In the model the continuation of the screening programme was set to 30 years (2009–2038). In this way the model enabled the evaluation of the screening as a public health intervention by estimating both lifetime costs and health benefits. Table 1 shows the data and sources used in the model. This study was approved by the Basque Country’s Ethics Committee.

**Table 1:** Sources of parameters used in the adaptation of the MISCAN-Colon model to the Basque population and screening programme

Data	Source
Demographics 2008	EUROSTAT – Basque Institute of Statistics
CRC incidence 2005–2008	Basque Cancer registry
Adenomas prevalence Base case	COLONPREV study [19]
Adenomas prevalence Low Prevalence case	[28, 29]
Costs screening	Basque CRC screening programme
Costs colonoscopy	Basque Health Service accounting system
Costs treatment	Basque Health Service accounting system
Utilities	[30]
Invitations 2009–2013	Basque CRC screening programme
Participation 2009–2012	Basque CRC screening programme
Test features 2011–2012	Basque CRC screening programme

*EUROSTAT* Statistical Office of the European Union, *CRC* colorectal cancer

As the MISCAN-Colon model was developed within the CISNET group, [26] it was first calibrated to CRC epidemiology and demographics of the United States population. Therefore, to use it for the Basque population, the age-specific CRC incidence by location (colon and rectum) and stage distribution were calibrated to the incidence data from the Basque Cancer Registry for the years 2005–2008, prior to the implementation of the CRC screening programme. Simultaneously, the MISCAN-Colon model was calibrated to the adenoma detection rates calculated for the Basque population from the COLONPREV study [20]. Separate models were built for men and women due to epidemiological differences in the natural history of CRC.

### Screening scenario simulated

Screening was simulated according to the design of the Basque colorectal cancer screening programme. All individuals within the programme’s age range biennially received an invitation letter that included a FIT. Delivering a sample to the assigned primary care health centre was necessary to have it analysed for microscopic bleeding. The cut-off was established at 20 μg/g faeces. Those participants with a positive test result were referred for colonoscopy. Furthermore, patients with at least one adenoma > 20 mm or five or more adenomas were recalled within a year for a surveillance colonoscopy and those with one adenoma > 10 mm or more than three adenomas or any adenomas with a villous component or high degree of dysplasia were invited for surveillance colonoscopy in three years, and all individuals with adenomas < 10 mm or 1 to 2 adenomas or tubular component or low degree of dysplasia were invited for regular FIT screening in 5 years. All recommendations were based on the European surveillance guidelines [13] adapted to the size-dependent classification in the model. A description of the surveillance protocol appears in the Additional file 2: Table S1. Participation rates both for FIT and colonoscopy were estimated for initial and successive invitations, depending on the individual’s age as determined by available data from the Basque CRC Screening Programme in the period 2009–2012 (Table 1).

### Test characteristics

Test characteristics were fitted to the positivity and detection rates of advanced neoplasia observed in the Basque programme between 2011 and 2012 (Additional file 2: Table S2). Advanced neoplasia included CRC and advanced adenomas, which were defined as adenomas ≥10 mm in size, with a 25% or greater villous component and/or high-grade dysplasia. The screening module was tested by reproducing invitations, participation and test results.

After the natural history of CRC without detection by screening was reproduced, the behaviour of CRC was reproduced according to the screening scenario, in which the impact of removing adenomas and anticipation of the CRC stage at diagnosis were considered. Those consequences were translated in quality-adjusted life years (QALY) gained and avoided costs in treatment.

### Costs and utilities

We applied the perspective of the Basque Health Service, which is responsible for delivering screening to the entire Basque population between 50 and 69 years old. Unit costs were obtained in Euros (Sep 2012: 1 Euro = 1.25 US dollars) from the accounting systems in the service. The resources assigned to each screened person were disaggregated by invitation (€6.06 including invitation letter, FIT and programme management resources), FIT analysis in participants (€0.99), primary care consultation in case of positive results (€78.00) and colonoscopies (€461.30 with polypectomy and €281.30 without polypectomy). We also applied an average cost of €5157.00 for complications related to colonoscopy. To calculate the costs of CRC treatment, we retrospectively collected stage and resource use from a sample of 529 patients [29]. For stages I to III the initial and follow-up costs were measured. The calculation of cost for stage IV disease combined generalized linear models to relate the cost to the duration of follow-up on the basis of parametric survival analysis. Unit costs were obtained from the analytical accounting system of the Basque Health Service. The sample included 110 cases in stage I, 171 in stage II, 158 in stage III and 90 in stage IV. The initial total cost ranged from €6968 for stage I to €12,765 for stage II and €13,075 for stage III. The estimation of the annual cost for follow-up care included computed tomography, colonoscopy, tests and external consultations, and an amount of €404 was rendered. For those patients in stage IV specific initial treatment cost was not considered, however, the cost for each year of follow-up was €24,255. The details of the generalized linear model are reported in the Additional file 2: Table S3.

As specific preference values for the different stages of CRC were not available for the Basque or Spanish population, we incorporated utilities that were already in the MISCAN-Colon model (Additional file 2: Table S4) [29]. We assumed a utility loss (i.e., a loss of QALY) equivalent to two full days of life per colonoscopy (0.0055 QALY) and two weeks of life per complication (0.0384 QALY). We also assigned a utility loss to each life-year with CRC care (Additional file 2: Table S4) [30].

### Economic evaluation

The economic evaluations included both the cost-effectiveness analysis and the budget impact analysis (BIA) of the programme from the perspective of the Basque Health System. The evaluation period was defined as 2009 through December 31, 2038. The incremental cost-effectiveness ratio (ICER) incorporated the additional QALY gained in the denominator and the additional costs incurred by the programme in the numerator [9]. We applied a multi-cohort approach by including all the cohorts in the programme during 30 years of implementation from its beginning in 2009. After this period, from 2039 onwards, the programme did not include more cohorts, but maintained the intervention for all individuals already included in the screening programme. The entire population had lifetime follow-up in order to capture the long-term impact. Costs and QALYs were discounted by 3%.

The microsimulation model was used simultaneously for BIA. The annual costs for CRC diagnosis and treatment in both the screened and unscreened populations from 2009 to 2038 were calculated in the model [16, 18]. Diagnostic resources included screening tests (FIT and diagnostic and surveillance colonoscopies) and clinical colonoscopies implemented in the reference hospital. It was not necessary to discount the costs because the BIA showed financial streams over time without aggregation [16].

### Sensitivity analysis

To assess the impact of model assumptions in our results, three main sensitivity analyses were carried out: 1) A scenario including a lower prevalence of adenomas based on the literature [31–40] was run in addition to the base case model; 2) Because individuals with a false negative test result have a higher than average probability of another false negative test result at a successive screening, we also developed a new scenario in which dependency of FIT results in sequential screening rounds was assumed (Additional file 2: Table S2) [41]; 3) The impact of increasing the cost of screening was also estimated.

## Results

### Goodness-of-fit

**Fig. 1:**
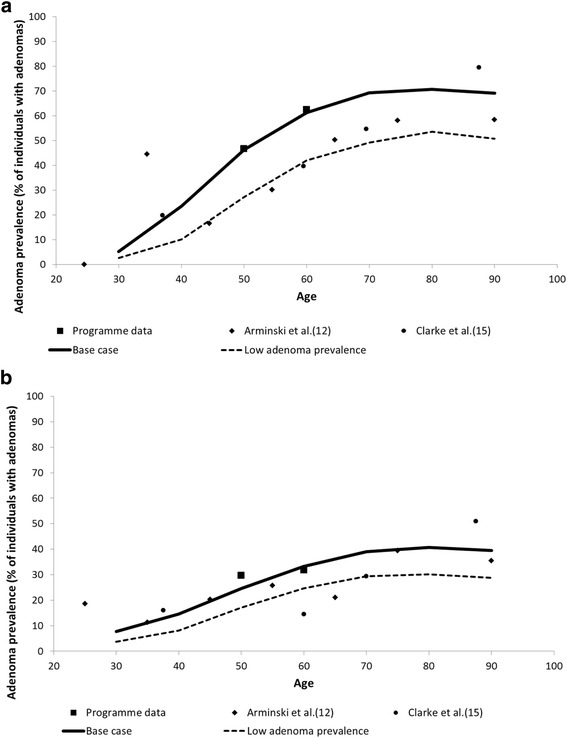
Adenomas Prevalence Observed in COLONPREV study and other published studies Versus Simulated by MISCAN-Colon (% of individuals with adenomas)*. **a**) Men; **b**) Women. *Observed results are only shown for the two largest studies on which the model has been calibrated. MISCAN-Colon has additionally been calibrated to 8 other autopsy studies

**Fig. 2:**
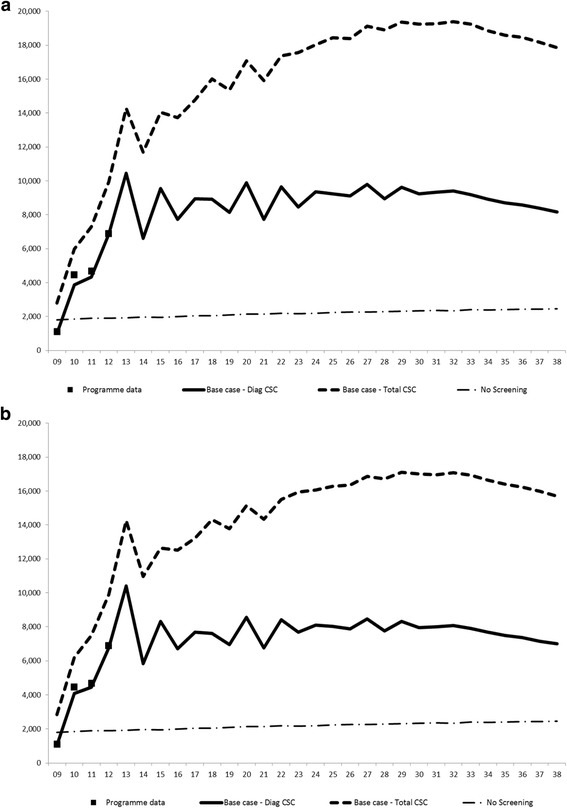
Predicted number of diagnostic and total colonoscopies needed each year in base-case scenario. **a**) Independent successive screening tests; **b**) Correlated successive screening tests. Diag: diagnostic; CSC: colonoscopies

Goodness-of-fit obtained in the MISCAN-Colon model adaptation for adenoma prevalence by age and gender, respectively, is shown in Fig. 1a and b and Model Appendix contain further validation details such as CRC incidence fitting. The model showed good concordance with the number of diagnostic colonoscopies observed in the Basque programme from 2009 to 2014 (Fig. 2a and b), validating the base scenario of high adenoma prevalence. Only in 2013 observed participation and positivity rates exceeded those simulated and the number of diagnostic colonoscopies carried out in the programme was also higher than forecasted. In 2014 the fit was better. Some other internal validation results, such as invitations and adherence to the programme and FIT characteristics in the period 2009–2014 were also used to validate model fitting and prediction behaviour (Additional file 2: Figure. S1 and Additional file 2: Figure S2). All these comparisons pointed to the good fit achieved in the adaptation of the MISCAN-Colon model to the Basque population and screening programme.

### Cost-effectiveness analysis

Without screening, the target Basque population in 2008 was expected to live on average 41.1 years (Table 2). After the implementation of the screening programme, that life expectancy increased by 29.3 days per person. This gain in terms of life-years involved an incremental effectiveness of 56,664.8 QALY (with 3% discount) due to screening. The total cost for screening, diagnostic follow-up, surveillance and treatment, during the applied time horizon, was €2057.2 million (Table 2). However, because screening resulted in a substantial reduction of colorectal cancer incidence, there was a large reduction in the costs of treating CRC (256.3 m€) and a net saving of €93.1 million (Table 2) compared to that in the unscreened population. Overall, these savings from treatment were larger than the costs for screening, and thus, the Basque screening programme was dominant compared to no screening.

**Table 2:** Sensitivity analysis for cost-effectiveness analysis of the implementation of colorectal cancer screening programme in the Basque population

		Screened population costs		Incremental costs		QALYs gained	ICER
Cost per invitation	Adenoma prevalence	Treatment cost	Total costs	Treatment cost	Total costs		
€6.06 per invitation	Base case						
	Men	1199.9	1317.0	−179.1	−81.7	37,132.5	Dominant
	Women	664.2	740.2	−77.1	−11.4	19,532.3	Dominant
	Total	1864.1	2057.2	−256.3	−93.1	56,664.8	Dominant
	Prevalence Bibliography						
	Men	1227.5	1318.9	−168.6	−97.2	36,616.9	Dominant
	Women	661.0	726.9	−77.6	−21.8	20,438.5	Dominant
	Total	1888.5	2045.7	− 246.2	−119.0	57,055.4	Dominant
€15.00 per invitation	Base case - Total	1864.1	2103.2	−256.3	−47.1	56,664.8	Dominant
€20.00 per invitation	Base case - Total	1864.1	2128.9	−256.3	−21.4	56,664.8	Dominant
€25.00 per invitation	Base case - Total	1864.1	2154.6	−256.3	4.2	56,664.8	74.1
€30.00 per invitation	Base case - Total	1864.1	2180.3	−256.3	30.0	56,664.8	529.4
€40.00 per invitation	Base case - Total	1864.1	2231.8	−256.3	81.5	56,664.8	1438.3
€50.00 per invitation	Base case - Total	1864.1	2283.2	−256.3	132.9	56,664.8	2345.4

*ICER* incremental cost effectiveness ratio

### Budget impact analysis

**Fig. 3:**
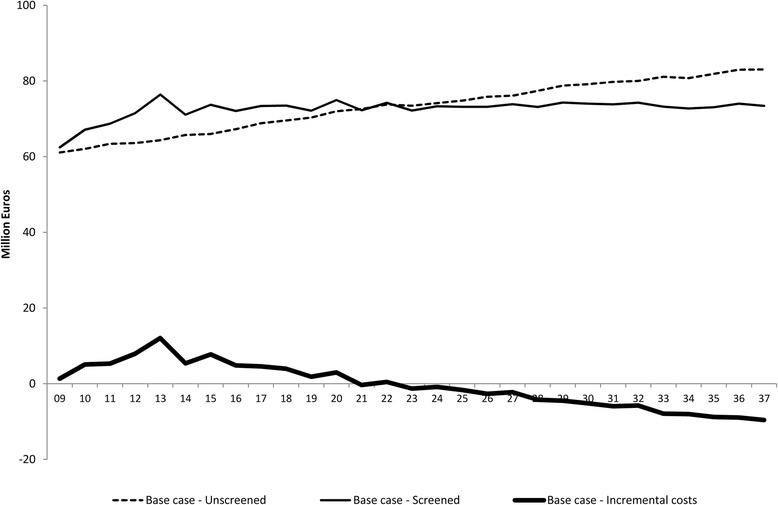
Budget impact analysis for base case scenario

Total and incremental costs related to screening, diagnosis and treatment of CRC in both scenarios (screened and unscreened) are displayed by year in Fig. 3. High screening costs due to the high number of positive test results for prevalent adenomas and cases of CRC were involved in the first four years after the introduction of screening until 2013, when 100% implementation was achieved. During the first five years of implementation, €69.2 million were necessary on average to annually fund the screening (Fig. 3 and Additional file 2: Table S5). The savings in the BIA appear in 2023, 10 years after the complete implementation of the programme. Even though the savings from screening were small at first, they increased as the impact of the programme became apparent. Figure 3 shows that the increase in the annual budget for the ageing unscreened population disappeared when screening was applied, and thus a stable annual budget of €73.4 million was achieved on average from year 2023 onwards.

In addition, the model predicted the number of colonoscopies during a 30-year period (Fig. 2), which reached a maximum in 2032 with 19,384. On average, the estimated annual number of colonoscopies (including diagnostic, surveillance and clinical colonoscopies) needed in the base case from 2029 through 2038 was 18,843 (52.5% surveillance colonoscopies).

### Sensitivity analysis

When a scenario with lower adenoma prevalence was set, the number of colonoscopies declined to 14,167 (Additional file 1: Figure. S3) and the net cost savings amounted to €119 million in the study period (Additional file 1: Figure. S4). Similarly, when the possibility of systematic false negative test results was included in the model, it was determined that fewer colonoscopies (16,605) than estimated for the base case were necessary and involved a savings of €95.1 million compared to the no-screening scenario. Finally, when unit costs for screening invitations were increased, the model showed that the screening programme would remain cost-saving if the cost of an invitation remained ≤20€ per invitation (€21.4 million saved) (Table 2).

## Discussion

This cost-effectiveness analysis featured the Basque CRC screening programme as a dominant intervention because it produced net cost savings and health benefits [9]. Budget analysis also showed a consistent savings after 10 years of its complete implementation, highlighting the affordability of the programme [16]. Both approaches to economic evaluation reveal that only a long follow-up is able to capture the late economic benefit of the screening based on the decrease in the number of cases of CRC in early and advanced stages and the consequent reduction in need for treatment. So far in Europe, no other population-based CRC screening programme has been evaluated with a comprehensive approach including both cost-effectiveness and budget impact analysis to prove the programme’s efficiency and affordability. Obviously, our results support the continuity of this preventive policy for CRC. Two literature reviews confirmed this finding, emphasising that colorectal cancer screening is cost effective or dominant compared with no screening [42, 43].

Public health programmes must be reappraised periodically to confirm that they have achieved planned results [44]. Modelling can also be useful in other countries to inform the population programme planning. Our sensitivity analysis showed the impact of the actual adenoma prevalence on the need of colonoscopies. A realistic estimation for the number of colonoscopies needed could be made based on the early report produced by the COLONPREV study [20].

Different strategies for CRC screening are supported by the evidence. As the Basque programme is based on FIT, our assessment cannot avoid comparison with an alternative approach based on colonoscopy as the first test, which is the norm in the United States. To make that comparison, however, the number of colonoscopies required for effective screening must be considered. Any evaluation must assess if a health system has the capacity to absorb such increased demand for screening colonoscopies. Typically, when the demand exceeds the capacity to deliver, the implementation of the programme is obstructed. Moreover, the required number of colonoscopies is directly related to the prevalence of adenomas in the specific population [14, 19]. In our study, this indicator was especially noteworthy because the adenoma prevalence in the Basque sample of the COLONPREV study yielded a higher number than that used in the MISCAN-Colon model [20]. The consequence of this finding could jeopardize the sustainability of the programme. The higher prevalence showed that 4200 more colonoscopies annually were necessary on average than were needed in the scenario based on a low prevalence. Both numbers were lower in the scenario in which dependency of FIT results in sequential screening rounds was assumed, that is, taking into account that individuals with a false negative test result have a higher than average probability of another false negative test result at a successive screening [41]. These predicted figures highlight the sustainability of the programme in operational terms, because the number of necessary diagnostic colonoscopies stabilized at approximately 8000, which was the number already being delivered. However, the maximum capacity of the Basque Health System to carry out screening colonoscopies has been reached. Thus, any other proposal for CRC screening that led to more colonoscopies would not be feasible with the currently available resources. As colonoscopies delivery become usually a bottleneck in the process of implementing CRC screening.

To explain the long-term cost savings, we need to underline that although the screening programme and the derived colonoscopies meant a significant expense, more than 90% of the total expenditure originated with treatment costs. To reach this conclusion, future results had to be predicted. So, although the number of required colonoscopies rose with increasing prevalence of adenomas, the impact on the total cost was limited [20, 31]. The total costs in the unscreened scenario grew steadily during the analysed years because the number of CRC cases in the Basque population rose with the aging of the baby boomers. The main component of those costs was the treatment costs that were proportional to the incidence. It is noteworthy that screening impact meant stabilization in total costs because the raw incidence was maintained and, therefore, the CRC age-adjusted rates decreased.

Decision-making on the basis of models is not without limitations. Although we used a model of high quality (MISCAN-Colon), which was adjusted to the epidemiology of the Basque population and the characteristics of the programme, model calibration was meant to force some parameters such as the classification of adenomas. The programme information system categories are risk-based, whereas the MISCAN-Colon model sorts them by size. The problem was resolved by reclassifying the adenomas found in the programme according to size. We understand that this adjustment did not involve any threat to the validity of the results, given the exhaustive process of calibration and validation. In addition, only costs related to the screening programme and CRC treatment were considered in the analysis; thus, we did not take into account the of cost of care of unrelated diseases that could appear due to increasing survival time. Including these costs could increase the final ICER and reduced the efficiency of the screening. Finally, the model did not include a probabilistic sensitivity analysis that is the norm in pharmacoeconomics, because of the difficulty of the computational burden in public health studies in which required dynamic models can include millions of individuals. However, the thorough calibration algorithm in MISCAN-Colon and the comprehensive internal and external validation warrant its robustness and, therefore, the reliability of the results.

Some of the differing results achieved in cost-effectiveness studies have been attributed to the dissimilarities in structure and parameters of the models applied to follow the entire life of the target population. The natural history of CRC as represented by the MISCAN-Colon model is shared by other CISNET partners, and its consistency has been fully proven [45, 46]. Moreover, the calibration of the MISCAN-Colon model to fit the epidemiology of CRC and adenoma prevalence in the Basque country was achieved by internal and external validation. As Patel et al. pointed out, preclinical time, screening unit costs in each setting or screening adherence, none of which is the same in all studies, can explain heterogeneous results [43]. Good intermediate indicators of programme performance, such as participation rate (64.3% in 2009–2011) and waiting time for colonoscopy (30 days), also show that the programme is on track and help to explain the final economic saving [10, 47]. Significantly, that conclusion was also valid when the unit cost of screening was doubled.

It is also important to put into context the relative costs of the screening programme, as they represented between 6% and 8% of the total cost, when we aggregated the costs of the whole follow-up. Furthermore, that percentage range will probably decrease, as treatment costs tend to grow as a consequence of the introduction of new drugs much more expensive than those currently on the market [48]. As oncology drugs become less cost effective because of rising prices over time [48], the role of preventive policies is enhanced. Thus, our results support the idea that reinforcement of screening seems a plausible future option. Consideration of inequalities in access to health care also sustains investments in screening, rather than treatment. Although differences by social class have been found in the screening programme, it is easier to provide disadvantaged groups with better access to screening services than to provide the entire population with the latest oncological drugs [49].

The BIA approach to economic evaluation is used less often than the cost-effectiveness design, but it is a complementary and useful tool to show financial streams over time. Usually the trade-offs between incurred and saved costs are shown with a short time horizon, but the BIA easily allows the application of a long follow-up to capture the full economic and health benefit of the screening programme. This is consistent with the natural history of CRC and has been underscored in the literature [5]. An extended period of time is required for the impact of polypectomies on avoided deaths and cancer treatments to manifest, and thus, the first years of the programme can be misleading because the change in natural history of CRC has not reached a steady state in terms of the population [47].

## Conclusions

We would like to emphasise that this evaluation reaffirms for decision-makers that the allocated resources for maintaining the programme are a worthwhile investment. This economic evaluation showed a screening intervention with a major health gain that also produced net savings when a long follow-up was used to capture the late economic benefit. Our results support the continuity of the Basque Colorectal Cancer Screnning Programme. Due to the actual adenoma prevalence reported in the COLONPREV study a realistic estimation of the future need of colonoscopies was necessary. The number of colonoscopies required was high but remain within the capacity of the Basque Health Service. So far in Europe, no other population Colorectal Cancer screening programme has been evaluated by budget impact analysis. Budget impact analysis highlighted the affordability of the programme showing consistent savings after 10 years of complete implementation.

## Additional files

Additional file 1: Model Appendix. Detailed description of the MISCAN-Colon model and the adaptation procedure. (PDF 2242 kb)

Additional file 2: **Table S1.** European guidelines for surveillance schedule after colonoscopy adapted to the size-dependent classification used in the model. **Table S2.** Test characteristics by sex fitted to the positivity and detection rates of advanced neoplasia observed in the Basque programme between 2011 and 2012. **Table S3.** Generalized liner model applied to estimate annual follow-up cost in patients with metastatic colorectal cancer. **Table S4.** Disutility values used in the model for colonoscopy, its complications and four cancer states depending on cancer detection stage. **Table S5.** Budget impact analysis of the colorectal cancer programme in million euros. **Figure S1.** Number of invited and participant population: Observed data in the Basque Screening Programme Versus Simulated by MISCAN-Colon. **Figure S2.** Number of detected adenomas and cases of cancer: Observed data in the Basque Screening Programme Versus Simulated by MISCAN-Colon. **Figure S3.** Predicted number of diagnostic and total colonoscopies needed each year in base-case and low prevalence scenarios. **Figure S4.** Budget impact analysis for base case and lower adenoma prevalence scenarios. (PDF 1299 kb)

## Abbreviations

BIA: Budget impact analysis
CISNET: Cancer Intervention and Screening Network
CRC: Colorectal Cancer
FIT: Faecal Immunochemical Testing
ICER: Incremental cost-effectiveness ratio
MISCAN: Microsimulation Screening Analysis
QALY: Quality adjusted life years
