# Supplementary material for: Cost-effectiveness and budget impact analyses of a colorectal cancer screening programme in a high adenoma prevalence scenario using MISCAN-Colon microsimulation model
Source: BMC Cancer. 2018 Apr 25;18:464. doi: 10.1186/s12885-018-4362-1 (PMC5918894; doi:10.1186/s12885-018-4362-1)

<u>MODEL APPENDIX</u> – MISCAN-Colon

General Model Structure

"MISCAN-Colon is a stochastic microsimulation model for colorectal cancer (CRC) programmed in Delphi (Borland Software Corporation, Scotts Valley, California, United States). It can be used to explain and predict trends in CRC incidence and mortality and to quantify the effects and costs of primary prevention of CRC, screening for CRC, and surveillance after polypectomy".

The term 'microsimulation' implies that individuals are moved through the model one at a time, rather than as proportions of a cohort. This allows future state transitions to depend on past transitions, giving the model a 'memory'. Furthermore, unlike most traditional Markov models, MISCAN-Colon does not use yearly transition probabilities; instead it generates durations in states, thereby increasing model flexibility and computational performance. The term 'stochastic' implies that the model simulates sequences of events by drawing from distributions of probabilities/ durations, rather than using fixed values. Hence, the results of the model are subject to random variation.

MISCAN-Colon consists of 3 modules: a demography module, natural history module, and screening module.

The Demography Module

Using birth- and life-tables representative for the 2008 Basque population, MISCAN-Colon draws a date of birth and a date of non-CRC death for each individual simulated. In MISCAN-Colon the maximum age an individual can achieve is exactly 100 years.

The Natural History Module

<u>Transitions</u>

As each simulated person ages, one or more adenomas may develop (Model Appendix Figure 1). These adenomas can be either progressive or non-progressive. Both progressive and non-progressive adenomas can grow in size from small (≤5mm), to medium (6-9mm), to large (≥10mm); however, only progressive adenomas can develop into preclinical cancer. A preclinical cancer may progress through stages I to IV; however, during each stage CRC may be diagnosed because of symptoms. The stage specific survival after the clinical diagnosis of colorectal cancer before age 75 was taken from the Comprehensive Cancer Centre South from 1989-2003 [50]. The survival for individuals aged 75 years and older was adjusted to fit the observed age-increasing mortality/incidence ratio. For individuals with synchronous CRCs at time of diagnosis, the survival of the most advanced cancer is used. The date of death for individuals with CRC is set to the earliest simulated death (either due to CRC or due to another cause (see: 'The demography module')).

Transition Probabilities and Durations in States

An individual's risk of developing adenomas depends on the individual's age and a personal risk index. As a result of the latter most individuals develop no adenomas, whilst some develop many. We assumed that the distribution of adenomas over the colon and rectum equals the distribution of cancers as observed in Basque cancer registry during the period 2005-2008 before the programme started. The age-specific onset of adenomas and the dispersion of the personal risk index were calibrated to data on the prevalence and multiplicity distribution of adenomas as observed in autopsy studies [31-40] and adjusted to the adenoma prevalence observed in the COLONPREV study (Model Appendix Figure 2A and 2B)[20]. The age-specific probability of adenoma-progressivity and the age- and localization-specific transition probabilities between preclinical cancer stages and between preclinical and clinical cancer stages were simultaneously calibrated to Basque cancer registry data on the age-, stage-, and

localization-specific incidence of CRC as observed during the period 2005-2008 before the introduction of screening (Model Appendix Figure 3A and 3B).

The average durations between the preclinical cancer stages were calibrated to the rates of screen-detected and interval cancers observed in randomized controlled trials evaluating screening using guaiac fecal occult blood tests [51-53]. This exercise has been described extensively in a publication by Lansdorp-Vogelaar and colleagues [23]. The average duration from the emergence of an adenoma (state 2) until progression into preclinical cancer (state 7) (i.e. the adenoma dwell-time) was calibrated to the rates of interval cancers (including surveillance detected cancers) observed in a randomized controlled trial evaluating once-only sigmoidoscopy screening (Model Appendix Figure 4) [54]. We assumed an equal overall dwell-time for adenomas developing into CRC from a medium size (30% of all CRCs) and from a large size (70% of all CRCs). All durations in the adenoma and preclinical cancer phase were drawn from exponential distributions. Durations within the adenoma phase and within the preclinical cancer phase were assumed to be perfectly correlated (i.e. if a small adenoma grows into a medium-sized adenoma rapidly, it will also grow into a large adenoma or develop into CRC rapidly); however, durations in the adenoma phase were assumed to be uncorrelated with durations in the preclinical cancer phase (i.e. a rapidly growing adenoma does not necessarily develop into a rapidly progressing cancer). The proportion of medium sized, non-progressive adenomas growing large and the average duration in the medium size, non-progressive adenoma state (state 5) were calibrated to size-specific adenoma detection rates observed in a Dutch randomized controlled trial on colonoscopy screening [55].

The Screening Module

Screening will alter some of the simulated life histories: Some cancers will be prevented by the detection and removal of adenomas; other cancers will be detected in an earlier stage with a more favorable survival. As the stage-specific survival of screen-detected CRC as observed in randomized

controlled trials on guaiac fecal occult blood testing was substantially more favorable than that of clinically detected CRC, even after correcting for lead-time bias [23], we assigned those screen-detected cancers that would have been clinically detected in the same stage the survival corresponding to a one stage less progressive cancer. Hence, a cancer screen-detected in stage II, that would also have been clinically diagnosed in stage II, is assigned the survival of a clinically diagnosed stage I cancer. The only exceptions were screen-detected stage IV cancers. These cancers were always assigned the survival of a clinically diagnosed stage IV cancer.

Integrating Modules

The demography module generates a date of birth and a date of non-CRC death for each individual simulated, creating a life-history without adenomas or CRC. In the first example shown in Model Appendix Figure 5A, the natural history module generates an adenoma. This adenoma progresses into preclinical cancer, which is diagnosed because of symptoms in stage II and results in CRC death before non-CRC death would have occurred. In the screening module a screening examination is simulated, indicated by the blue arrow. During this examination the adenoma is detected, and as a result both CRC and CRC death are prevented. Hence, in this case screening prolongs life by the amount indicated by the green arrow. In the second example (Model Appendix Figure 5B) the patient also develops an adenoma, and although this adenoma does progress into preclinical cancer, this patient would never have been diagnosed with CRC in a scenario without screening. However, during the screening examination simulated in the screening module, again indicated by the blue arrow, CRC is screen-detected in stage I. Hence, in this patient screening results in over-diagnosis of CRC: It detects a cancer that would never have been diagnosed in a scenario without screening. Hence, screening does not prolong life, but it does result in additional LYs with CRC care (over-treatment) as indicated by the red arrow.

The Screening Parameters

We assumed a cecal intubation rate of 95% [56-58]. The sensitivity of colonoscopy for each lesion within realized reach was based on back-to-back colonoscopy studies: 75% in adenomas less than or equal to 5 mm, 85% in adenomas 6–9 mm, and 95% in adenomas greater than or equal to 10 mm and cancers [58]. After a positive test, all lesions are removed within a short time. The percentage of the population without adenomas or cancer but with hyperplastic polyps, lipomas, or other lesions that lead to polypectomy and pathology after colonoscopy has been estimated from Kaiser data:[60] 10%. This percentage was assumed to be independent of the screening round.

The stage-specific survival of patients with screen-detected cancer was based on a previous analysis calibrating on three large randomized FOBT-trials [23], and was more favourable than the survival after diagnosis in the same stage without screen-detecting. Removal of an adenoma always prevents development of any subsequent cancer that may have arisen from this adenoma. Risks of complications reported in organized screening programmes [61-63] are lower than those reported for general practice colonoscopies [64,65]. The major complications of colonoscopy are perforations (which can occur with or without polypectomy), serosal burns, bleeds requiring transfusion and bleeds not requiring transfusion [61-65]. We estimated a rate of death of 1 per 30,000 for colonoscopies with a polypectomy [66,67].

REFERENCES

50. Lemmens V, van Steenbergen L, Janssen-Heijnen M, Martijn H, Rutten H, Coebergh JW. Trends in colorectal cancer in the south of the Netherlands 1975-2007: rectal cancer survival levels with colon cancer survival. Acta Oncol. 2010;49(6):784-96.
51. Hardcastle JD, Chamberlain JO, Robinson MH, et al. Randomised controlled trial of faecal-occult-blood screening for colorectal cancer. Lancet. 1996; 348: 1472-1477.
52. Jorgensen OD, Kronborg O, Fenger C. A randomized study of screening for colorectal cancer using faecal occult blood testing: results after 13 years and 7 biennial screening rounds. Gut. 2002; 50: 29-32.
53. Mandel JS, Church TR, Ederer F, Bond JH. Colorectal cancer mortality: effectiveness of biennial screening for fecal occult blood. J Natl Cancer Inst. 1999; 91: 434-437.
54. Atkin WS, Cook CF, Cuzick J, Edwards R, Northover JM, Wardle J, UK Flexible Sigmoidoscopy Screening Trial Investigators. Single flexible sigmoidoscopy screening to prevent colorectal cancer: baseline findings of a UK multicentre randomised trial. *Lancet*. 2002;359(9314):1291-1300.
55. Stoop EM, de Haan MC, de Wijkerslooth TR, Bossuyt PM, van Ballegooijen M, Nio CY, et al. Participation and yield of colonoscopy versus non-cathartic CT colonography in population-based screening for colorectal cancer: a randomised controlled trial. *Lancet Oncol*. 2012;13(1):55-64.
56. Aslinia F, Uradomo L, Steele A, Greenwald BD, Raufman JP. Quality assessment of colonoscopic cecal intubation: an analysis of 6 years of continuous practice at a university hospital. *Am J Gastroenterol*. 2006;101(4):721-31.
57. Cotterill M, Gasparelli R, Kirby E. Colorectal cancer detection in a rural community. Development of a colonoscopy screening program. *Can Fam Physician*. 2005;51:1224-8.
58. Rex DK, Bond JH, Winawer S, Levin TR, Burt RW, Johnson DA, et al. Quality in the technical performance of colonoscopy and the continuous quality improvement process for colonoscopy: recommendations of the U.S. Multi-Society Task Force on Colorectal Cancer. *Am J Gastroenterol*. 2002;97(6):1296-308.
59. van Rijn JC, Reitsma JB, Stoker J, Bossuyt PM, van Deventer SJ, Dekker E. Polyp miss rate determined by tandem colonoscopy: a systematic review. *Am J Gastroenterol*. 2006;101(2):343-50.
60. Levin TR, Palitz A, Grossman S, Conell C, Finkler L, Ackerson L, et al. Predicting advanced proximal colonic neoplasia with screening sigmoidoscopy. *Jama*. 1999;281(17):1611-7.

61. Lieberman DA, Weiss DG, Bond JH, Ahnen DJ, Garewal H, Chejfec G. Use of colonoscopy to screen asymptomatic adults for colorectal cancer. Veterans Affairs Cooperative Study Group 380. *N Engl J Med*. 2000;343(3):162-8.
62. Pox C, Schmiegel W, Classen M. Current status of screening colonoscopy in Europe and in the United States. *Endoscopy*. 2007;39(2):168-73.
63. Regula J, Rupinski M, Kraszewska E, Polkowski M, Pachlewski J, Orlowska J, et al. Colonoscopy in colorectal-cancer screening for detection of advanced neoplasia. *N Engl J Med*. 2006;355(18):1863-72.
64. Levin TR, Conell C, Shapiro JA, Chazan SG, Nadel MR, Selby JV. Complications of screening flexible sigmoidoscopy. *Gastroenterology*. 2002;123(6):1786-92.
65. Levin TR, Zhao W, Conell C, Seeff LC, Manninen DL, Shapiro JA, et al. Complications of colonoscopy in an integrated health care delivery system. *Ann Intern Med*. 2006;145(12):880-6.
66. Warren JL, Klabunde CN, Mariotto AB, Meekins A, Topor M, Brown ML, et al. Adverse events after outpatient colonoscopy in the Medicare population. *Ann Intern Med*. 2009;150(12):849-57, W152.
67. Gatto NM, Frucht H, Sundararajan V, Jacobson JS, Grann VR, Neugut AI. Risk of perforation after colonoscopy and sigmoidoscopy: a population-based study. *J Natl Cancer Inst*. 2003;95(3):230-6.

Model Appendix Figure 1. An Overview of the Natural History Module of MISCAN-Colon.

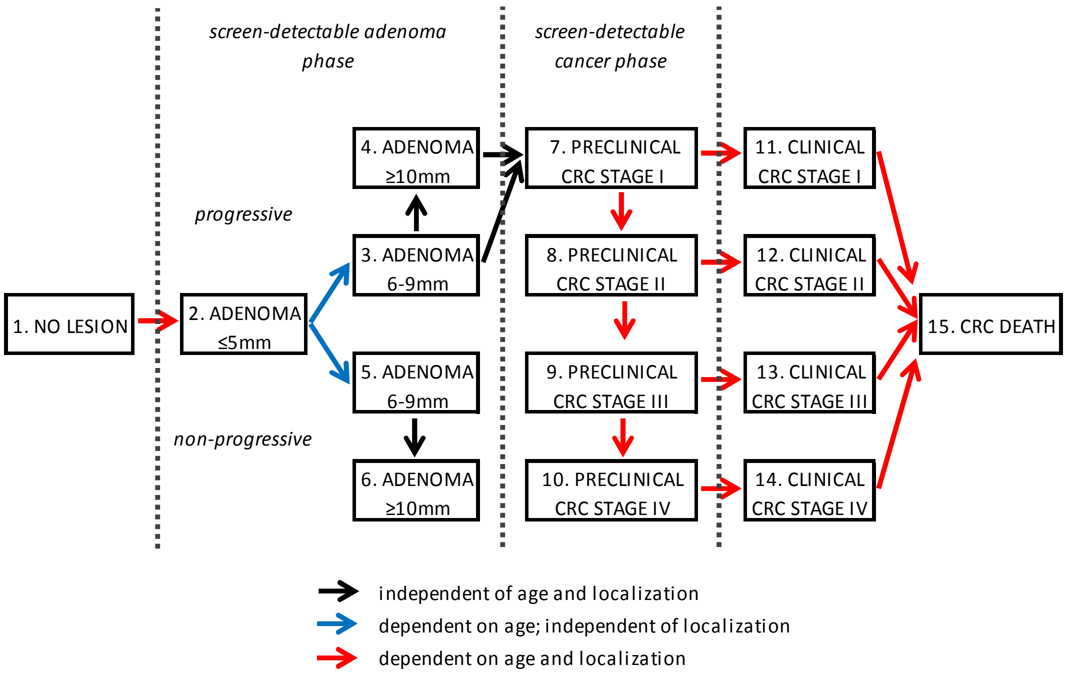

*Model Appendix Figure 2A. Adenomas Prevalence in men Observed in COLONPREV study and other published studies Versus Simulated by MISCAN-Colon(% of individuals with adenomas).**

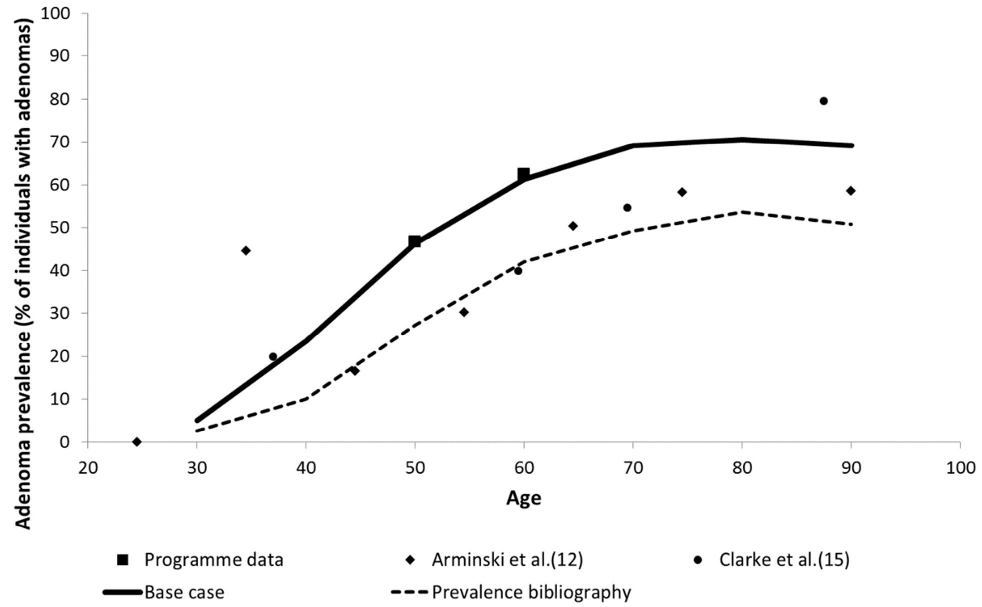

*Observed results are only shown for the two largest studies on which the model has been calibrated. MISCAN-Colon has additionally been calibrated to 8 other autopsy studies.

*Model Appendix Figure 2B. Adenomas Prevalence in women Observed in COLONPREV study and other published studies Versus Simulated by MISCAN-Colon(% of individuals with adenomas).**

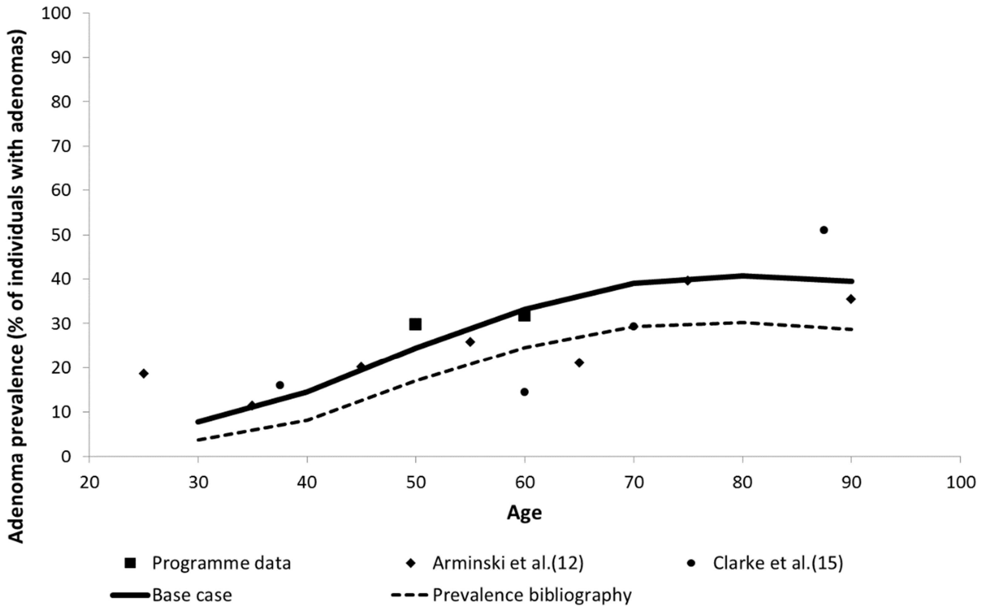

*Observed results are only shown for the two largest studies on which the model has been calibrated. MISCAN-Colon has additionally been calibrated to 8 other autopsy studies.

Model Appendix Figure 3A. CRC Incidence in men Observed Before the Introduction of Screening Versus Simulated by MISCAN-Colon (total (A), stage I CRC (B), stage II CRC (C), stage III CRC (D), stage IV CRC (E); cases per 100,000 person years).

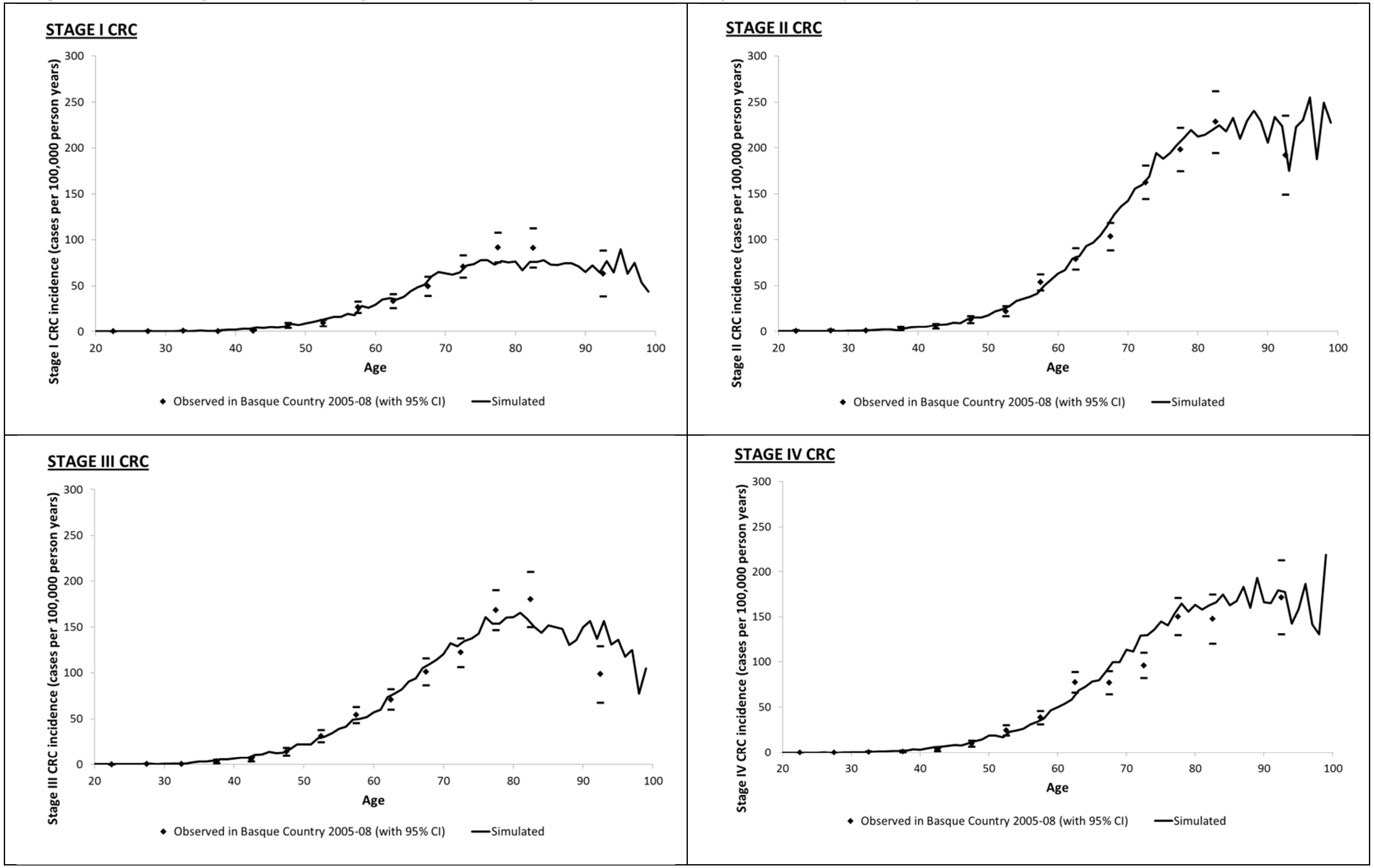

Model Appendix Figure 3B. CRC Incidence Observed Before the Introduction of Screening Versus Simulated by MISCAN-Colon (total (A), stage I CRC (B), stage II CRC (C), stage III CRC (D), stage IV CRC (E); cases per 100,000 person years).

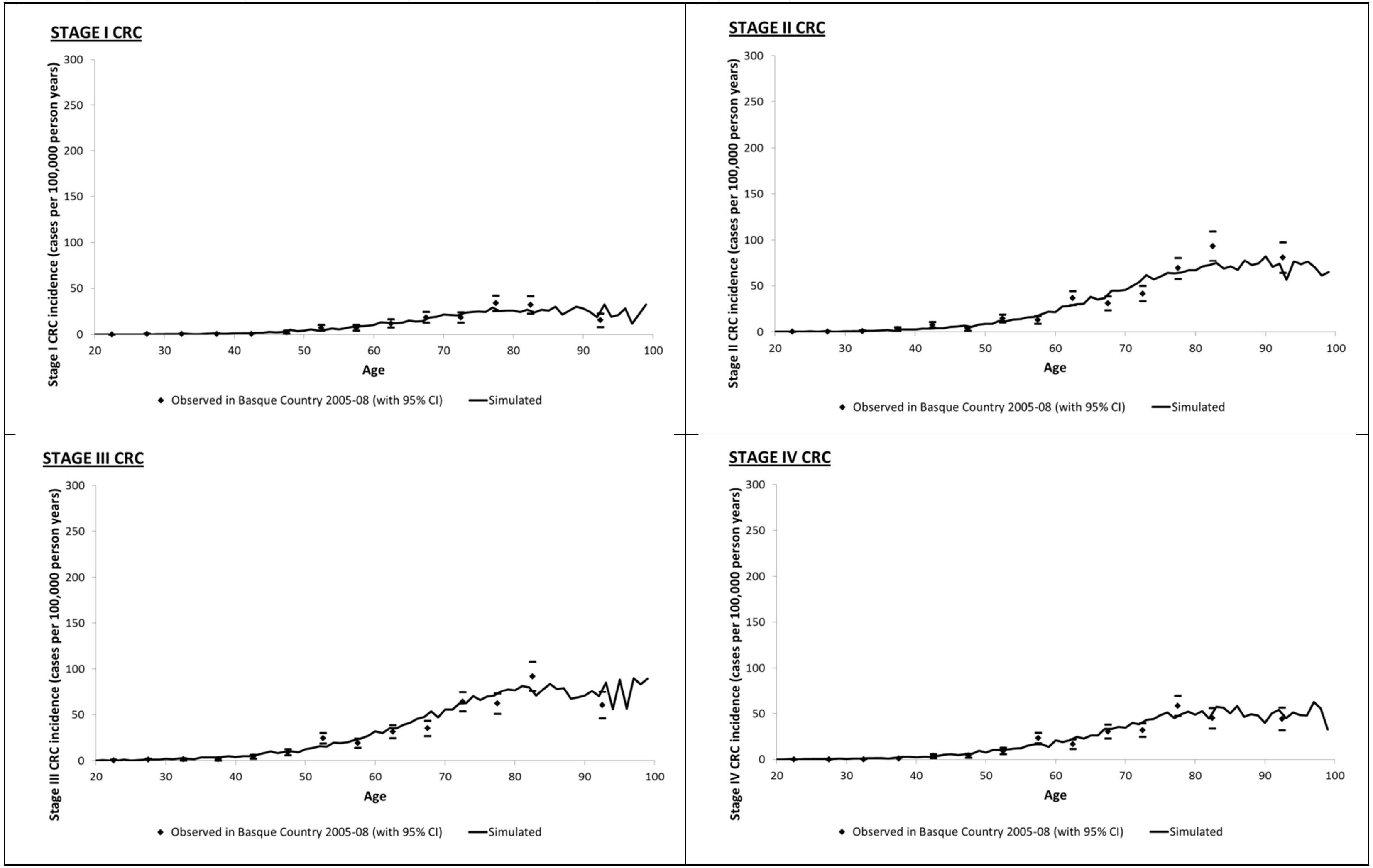

Model Appendix Figure 4A. Distal CRC Incidence Observed in the Intervention Group of the UK Flexible Sigmoidoscopy Trial Versus Simulated by MISCAN-Colon (cases per 100,000 person years, per year of follow-up).

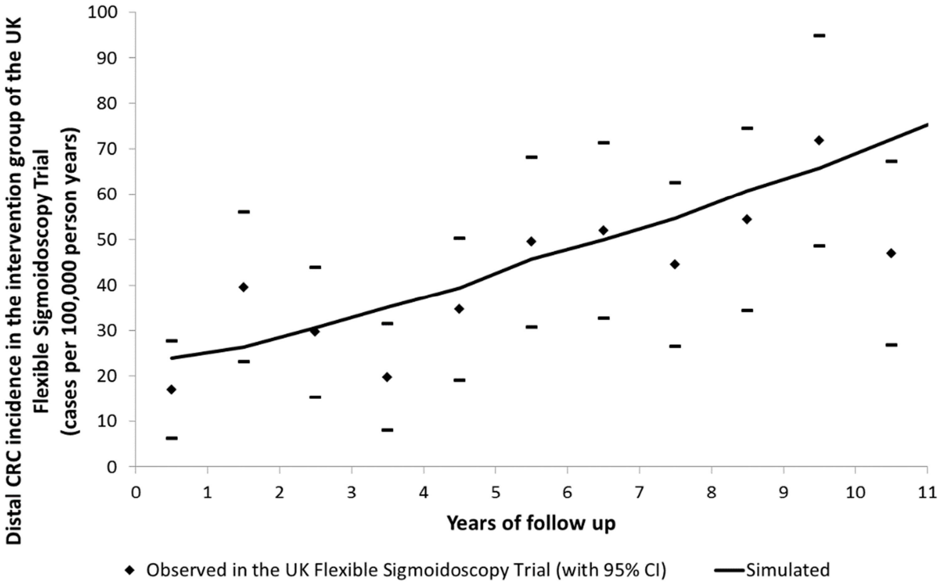

Model Appendix Figure 4B. Distal CRC Incidence Observed in the Intervention Group of the UK Flexible Sigmoidoscopy Trial Versus Simulated by MISCAN-Colon (cases per 100,000 person years, cumulative).

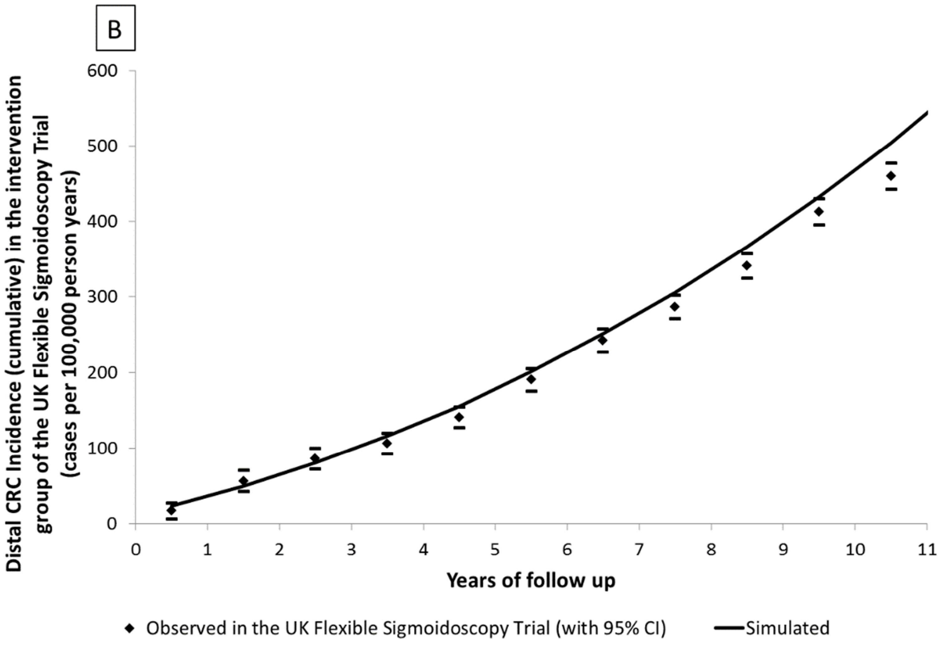

Model Appendix Figure 5A. Integrating Modules: Two example Patients.

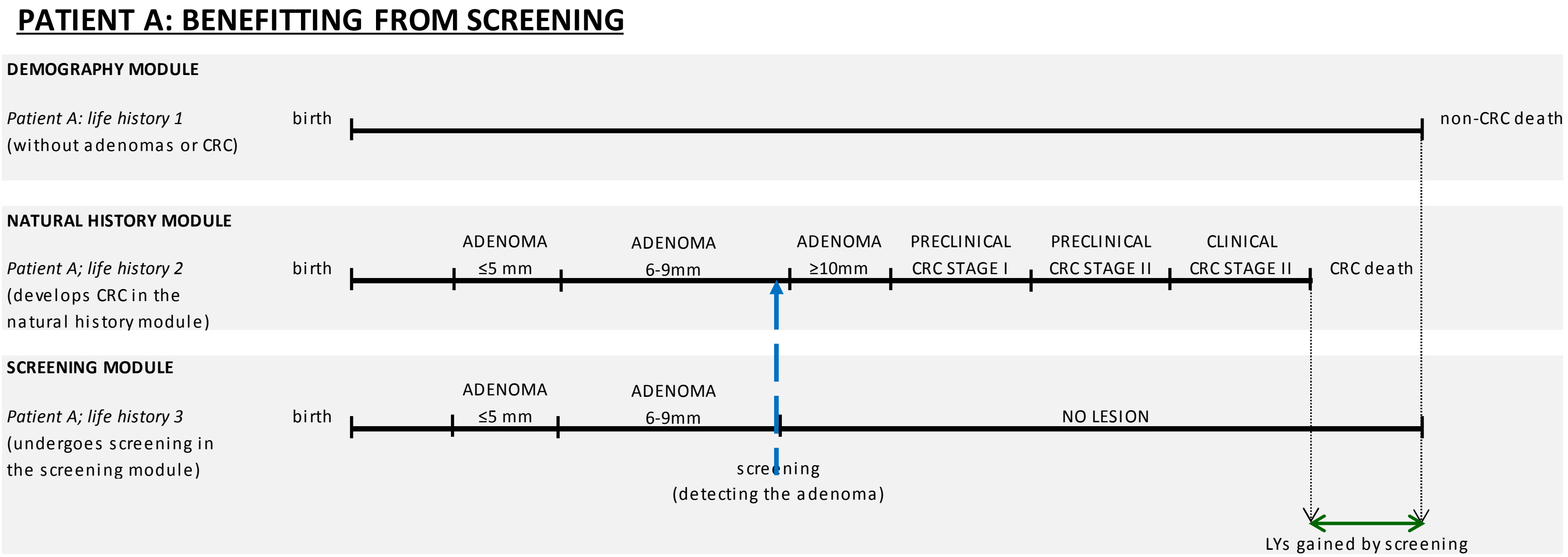

Model Appendix Figure 5B. Integrating Modules: Two example Patients.

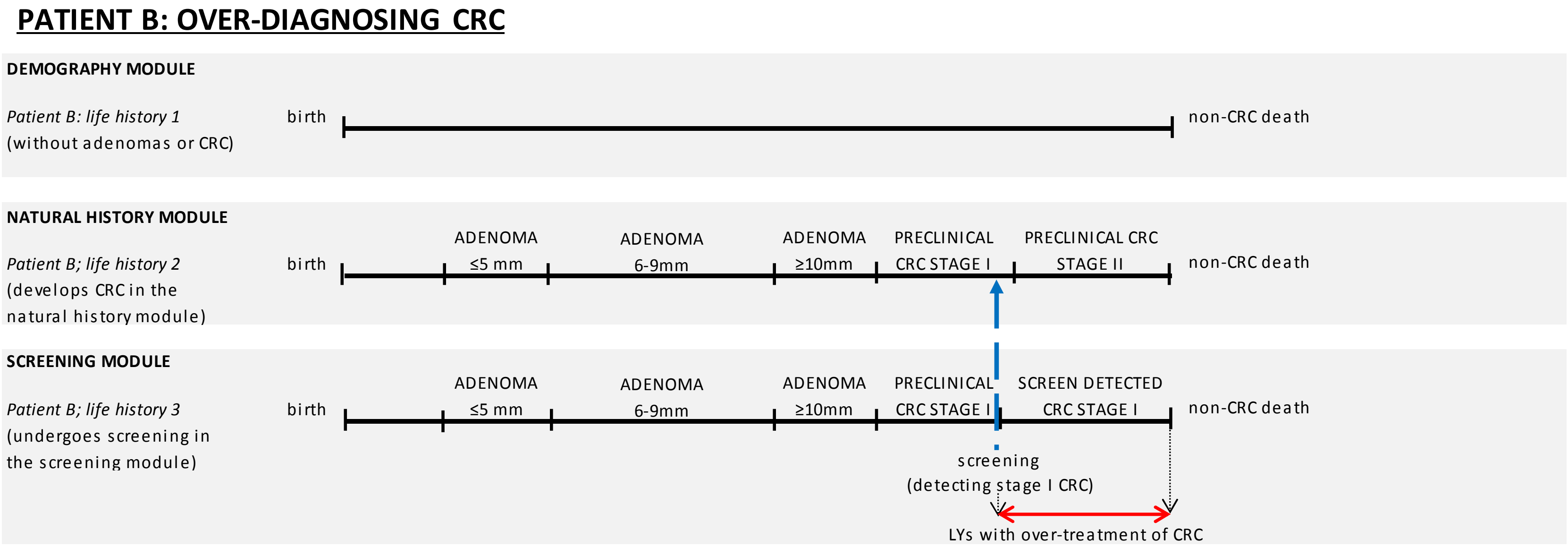

Supplement: Supplementary file 1 — Model Appendix. Detailed description of the MISCAN-Colon model and the adaptation procedure. (PDF 2242 kb) [file 12885_2018_4362_MOESM1_ESM.pdf]
