# Supplementary material for: Cost-effectiveness and budget impact analyses of a colorectal cancer screening programme in a high adenoma prevalence scenario using MISCAN-Colon microsimulation model
Source: BMC Cancer. 2018 Apr 25;18:464. doi: 10.1186/s12885-018-4362-1 (PMC5918894; doi:10.1186/s12885-018-4362-1)

Table S1. European guidelines for surveillance schedule after colonoscopy adapted to the size-dependent classification used in the model.

Category	Colonoscopy result	Next test	Time
Normal	No adenoma detection (age 50-59)	FIT (programme)	10 years
Low-risk adenoma	1-2 adenomas smaller than 1 cm (age 50-64)	FIT (programme)	5 years
Medium-risk adenoma	>2 adenomas smaller than 1 cm (age 50-71)	Surveillance colonoscopy	3 years
High-risk adenoma	1 adenoma larger than 1 cm (age 50-73)	Surveillance colonoscopy	1 year

FIT = faecal immunological testing

Table S2. Test characteristics by sex fitted to the positivity and detection rates of advanced neoplasia observed in the Basque programme between 2011 and 2012.

	FIT in Men	FIT in Women
Base case		
Normal	0.0396	0.0297
Adenomas < 5 mm	0.000	0.000
Adenomas 6-9 mm	0.043	0.050
Adenomas >= 10 mm	0.216	0.113
CRC type A	0.706	0.720
CRC type B	0.341	0.357
Correlated test results		
Normal*	0.0429	0.0317
Adenomas < 5 mm	0.000	0.000
Adenomas 6-9 mm**	0.047	0.049
Adenomas >= 10 mm**	0.228	0.116
CRC type A	0.706	0.719
CRC type B	0.340	0.355

*Probability of systematic false positive result. 0.0127. [40] **Probability of systematic negative result in adenomas: 0.7313 (6-9 mm), 0.2602 (>10 mm) [40]. FIT = faecal immunological testing; CRC = colorectal cancer

Table S3. Generalized liner model applied to estimate annual follow-up cost in patients with metastatic colorectal cancer.

Parameters	Coefficients	p-value
Constant	5470.40	< 0.001
Follow-up (years)	3366.90	< 0.001
Estimated mean cost per year	24254.90	

*Familia: Gamma; Función link: Potencia 0.9.

Table S4. Disutility values used in the model for colonoscopy, its complications and four cancer states depending on cancer detection stage.

		Disutilities
Colonoscopy		0.0055
Complication		0.0384
CRC in Stage I	Initial phase	0.12
	Follow-up	0.05
	Terminal phase by CRC	0.70
	Terminal phase by other causes	0.05
CRC in Stage II	Initial phase	0.18
	Follow-up	0.05
	Terminal phase by CRC	0.70
	Terminal phase by other causes	0.05
CRC in Stage III	Initial phase	0.24
	Follow-up	0.24
	Terminal phase by CRC	0.70
	Terminal phase by other causes	0.24
CRC in Stage IV	Initial phase	0.70
	Follow-up	0.70
	Terminal phase by CRC	0.70
	Terminal phase by other causes	0.70

CRC = colorectal cancer

Table S5. Budget impact analysis of the colorectal cancer programme in million euros.

	Screened population				Unscreened population		
Year	Screening	Clinical diagnosis	Treatment	Total costs	Clinical diagnosis	Treatment	Total costs*
2009	0.68	0.85	60.9	62.5	0.9	60.3	61.1
2010	2.75	0.86	63.5	67.1	0.9	61.2	62.0
2011	3.37	0.86	64.5	68.7	0.9	62.5	63.4
2012	5.17	0.83	65.5	71.5	0.9	62.7	63.6
2013	7.93	0.81	67.7	76.4	0.9	63.4	64.4
2014	5.75	0.80	64.6	71.1	0.9	64.8	65.7
2015	7.54	0.78	65.4	73.7	0.9	65.1	66.0
2020	8.69	0.77	65.5	75.0	1.0	71.0	72.0
2025	9.02	0.74	63.4	73.2	1.1	73.8	74.8
2030	9.30	0.75	63.9	74.0	1.1	78.1	79.2
2035	8.87	0.75	63.5	73.1	1.1	80.7	81.9
2038	8.43	0.75	63.7	72.9	1.2	81.7	82.9

*No screening costs in the unscreened population

Figure S1. Number of invited and participant population: Observed data in the Basque Screening Programme Versus Simulated by MISCAN-Colon.

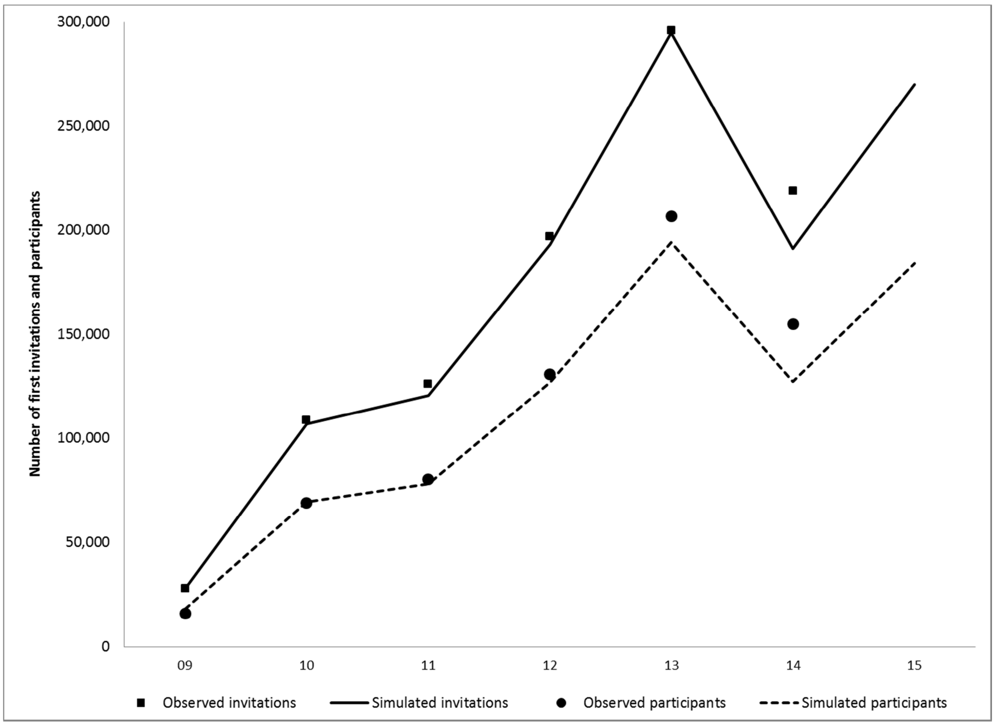

Figure S2. Number of detected adenomas and cases of cancer: Observed data in the Basque Screening Programme Versus Simulated by MISCAN-Colon.

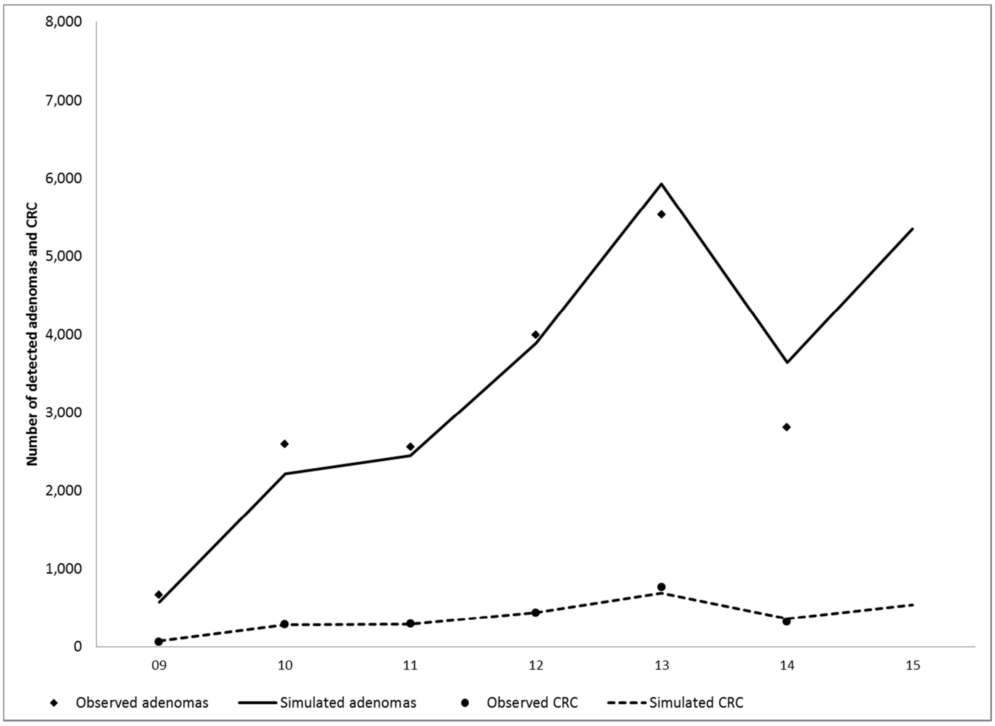

Figure S3: Predicted number of diagnostic and total colonoscopies needed each year in base-case and low prevalence scenarios.

A) Independent successive screening tests

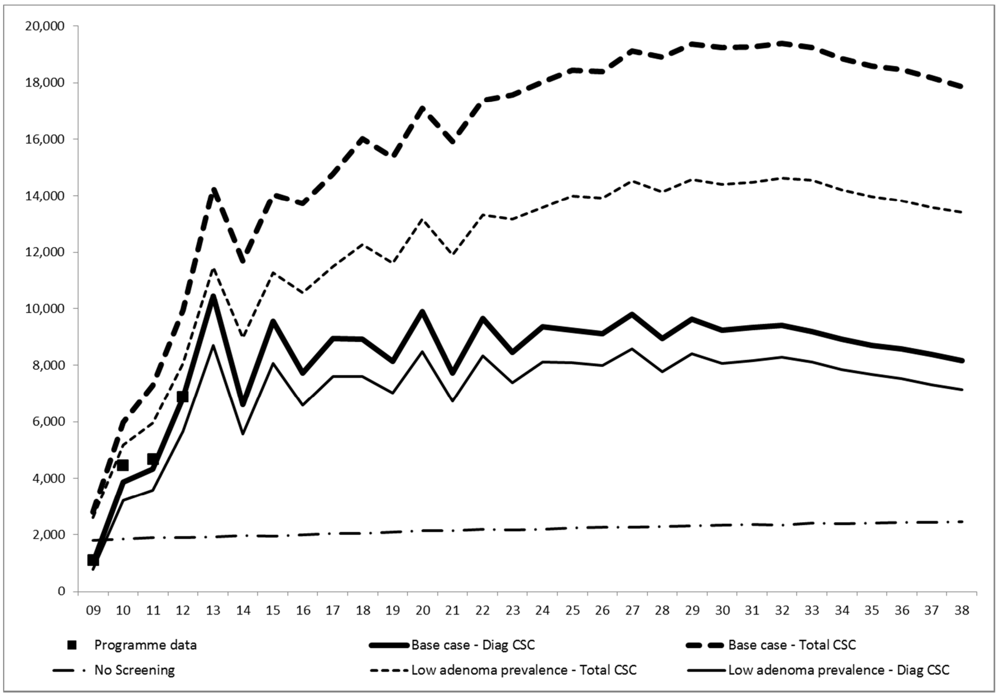

B) Correlated successive screening tests.

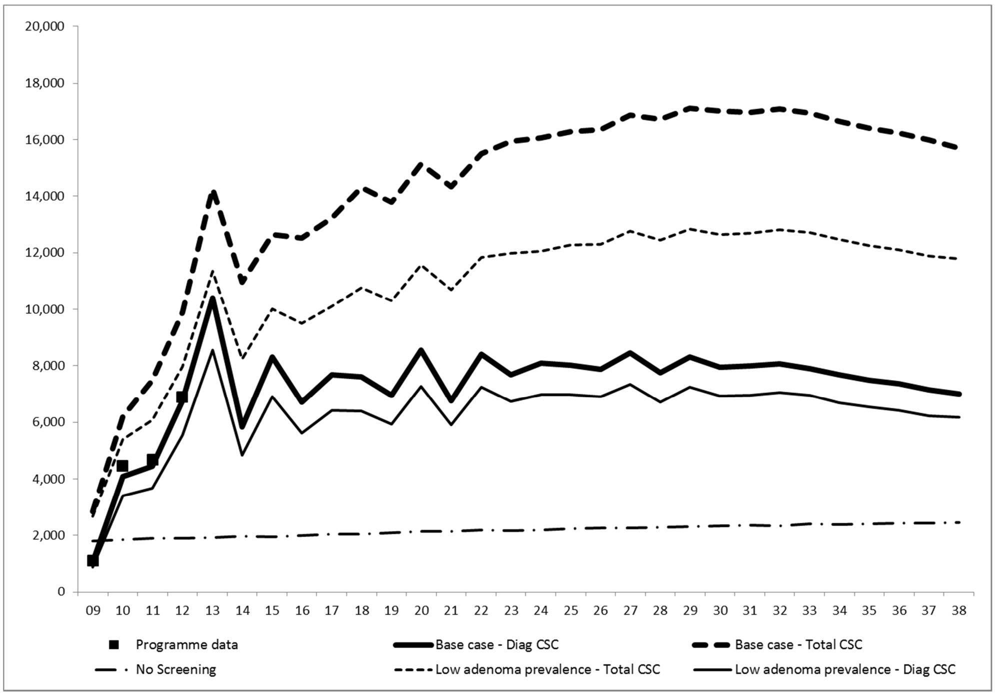

Figure S4. Budget impact analysis for base case and lower adenoma prevalence scenarios.

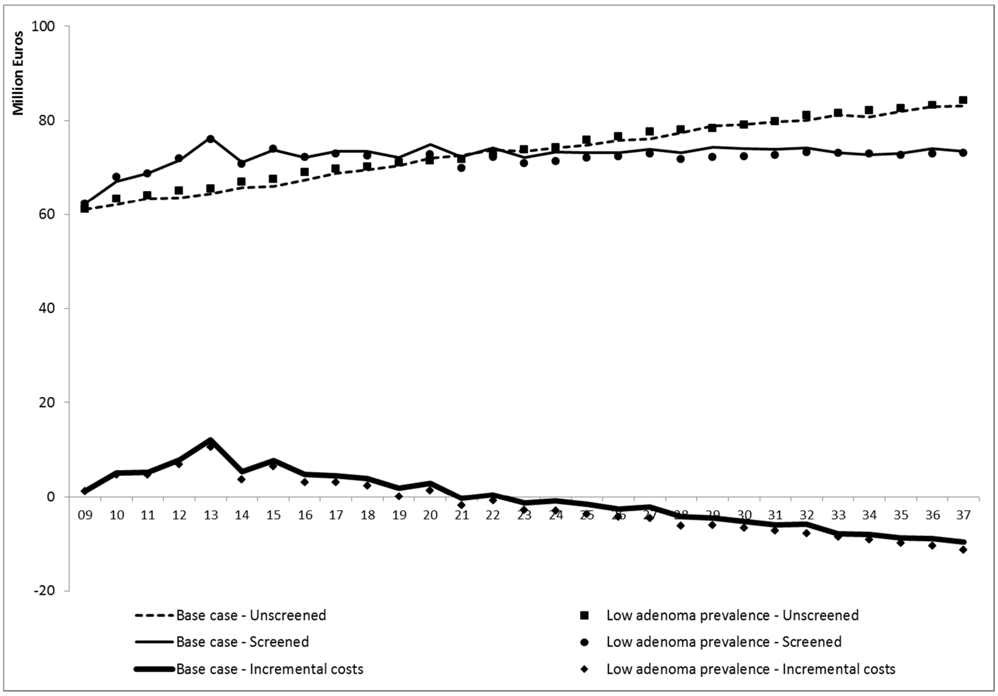

Supplement: Supplementary file 2 — Table S1. European guidelines for surveillance schedule after colonoscopy adapted to the size-dependent classification used in the model. Table S2. Test characteristics by sex fitted to the positivity and detection rates of advanced neoplasia observed in the Basque programme between 2011 and 2012. Table S3. Generalized liner model applied to estimate annual follow-up cost in patients with metastatic colorectal cancer. Table S4. Disutility values used in the model for colonoscopy, its complications and four cancer states depending on cancer detection stage. Table S5. Budget impact analysis of the colorectal cancer programme in million euros. Figure S1. Number of invited and participant population: Observed data in the Basque Screening Programme Versus Simulated by MISCAN-Colon. Figure S2. Number of detected adenomas and cases of cancer: Observed data in the Basque Screening Programme Versus Simulated by MISCAN-Colon. Figure S3. Predicted number of diagnostic and total colonoscopies needed each year in base-case and low prevalence scenarios. Figure S4. Budget impact analysis for base case and lower adenoma prevalence scenarios. (PDF 1299 kb) [file 12885_2018_4362_MOESM2_ESM.pdf]
